# Elucidating the Disparate Impact of Purification Levels on *Lycium barbarum* Phenolic Extracts: Chemical Composition and Hypoglycemic Enzyme Inhibition

**DOI:** 10.1002/fsn3.71077

**Published:** 2025-11-11

**Authors:** Qian Ma, Linwu Ran, Lu Lu, Lutao Zhang, Kiran Thakur, Zhaojun Wei, Jia Mi, Yamei Yan

**Affiliations:** ^1^ Institute of Wolfberry Science, Ningxia Academy of Agriculture and Forestry Sciences Yinchuan Ningxia People's Republic of China; ^2^ School of Food and Biological Engineering Hefei University of Technology Hefei People's Republic of China; ^3^ School of Public Health Ningxia Medical University Yinchuan People's Republic of China; ^4^ School of Biological Science and Engineering North Minzu University Yinchuan People's Republic of China

**Keywords:** *α*‐amylase, *α*‐glucosidase, isolation and purification, *Lycium barbarum*, phenolic amine compounds

## Abstract

Our study emphasizes the hypoglycemic potential of phenolic compounds extracted and purified from *Lycium barbarum* L. (*L. barbarum*) fruits. *L. barbarum* polyphenol extracts were purified using D101 macroporous resin, Sephadex LH‐20 dextran gel, and C18 chromatographic column, with their *α*‐amylase/*α*‐glucosidase inhibitory activities assessed across purity grades. The results revealed 78 chemical components in the crude extract (C1), with 34 phenolic compounds identified after purification with macroporous resin (D1), and 25 phenolic compounds detected after further purification with Sephadex LH‐20 gel (S1). Each purification step selectively altered the composition and content of the compounds, with substantial reductions observed in saponins and phenolic acids. Lycibarbarphenylpropanoid I (P1) was identified and characterized as a novel compound. Fraction C1 demonstrated the highest *α*‐amylase inhibitory activity, while S1 exhibited the highest *α*‐glucosidase inhibition. This suggests that different purification levels can enhance specific bioactivities responsible for hypoglycemic effects. The influence of various components on hypoglycemic enzyme activity varies significantly, suggesting the need for deeper exploration. This study presents critical insights into the phenolic extracts from *L. barbarum* fruits, and their hypoglycemic activity offers significant understanding of the application of these bioactive compounds.

## Graphical abstract

The objectives of this study were to emphasize the hypoglycemic potential of phenolic compounds extracted and purified from *Lycium barbarum* fruits. The results revealed 78 chemical components in the crude extract (C1), with 34 phenolic compounds were identified after purification with macroporous resin (D1), and 25 phenolic compounds were detected after further purification with Sephadex LH‐20 gel (S1). Each purification step selectively altered the composition and content of the compounds, with substantial reductions observed in saponins and phenolic acids. Lycibarbarphenylpropaoid I (P1) was identified and analyzed as a novel compound. C1 demonstrated strong α‐amylase inhibitory activity, while S1 exhibited excellent α‐glucosidase inhibition. This suggests that different purification levels can enhance specific bioactivities responsible for hypoglycemic effects. This study presents critical insights into the phenolic extracts from Ningxia *L. barbarum* fruits and their hypoglycemic activity offers significant understanding to the application of these bioactive compounds.

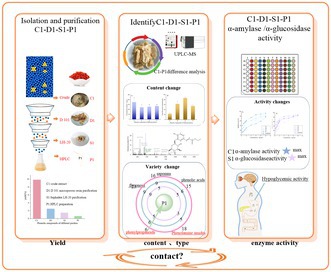

## 1 Introduction

Phenolic compounds, characterized by the hydroxyl (‐OH) functional groups, are widely distributed in natural products and are critical bioactive components in *L. barbarum* (Blesso and Christopher 2024; Lucia et al. 2020). Alongside polysaccharides—another major class of hypoglycemic compounds in *L. barbarum* (Ma, Sun, et al. 2023). They exhibit diverse pharmacological activities, including hypoglycemic (Donno et al. 2016), antioxidant (Ma, Wang, et al. 2023; Ma, Sun, et al. 2023), anti‐inflammatory (Ma, Wang, et al. 2023; Ma, Sun, et al. 2023), and anti‐tumor (Wu et al. 2016) effects, making them highly significant to human health. As a geo‐authentic Chinese medicinal material and dietary supplement (Niu et al. 2022), *L. barbarum* is a plant rich in various functional components such as polysaccharides and polyphenols, and it has good hypoglycemic activity. Among them, polyphenols are one of its active components, which have attracted extensive attention. Macroporus D101 resin and Sephadex LH‐20 are extensively used for the separation and purification of phenolic compounds due to their porous structure, stable physical and chemical properties, and the combination of adsorption and screening properties (Wang and Wang 2019). Studies have reported the selective influence of these techniques on the composition and content of bioactive compounds. For instance, studies on pomegranate peel extract have demonstrated that ultrafiltration and nanofiltration processes can significantly alter the content and types of phenolic and flavonoid compounds, thereby influencing their antioxidant and antidiabetic activities (He et al. 2010). In the context of *L. barbarum* , advanced analytical techniques, such as UPLC‐Q‐Orbitrap MS (Zhang et al. 2018), enable rapid identification and quantification of phenolic compounds. Additionally, UPLC‐MS and FIMS fingerprints have been utilized to distinguish *L. barbarum* varieties and origins (Lu et al. 2014). Furthermore, HPLC‐MS/MS has found 40 phenolic compounds in dried *L. barbarum* (Shang et al. 2022). Despite advancements in identifying and quantifying phenolic compounds, research on the relationship between the content, type, and biological activity of these compounds at different purification levels remains limited. Hyperglycemia, a hallmark of diabetes and other metabolic disorders (Echouffo et al. 2017), poses a significant threat to human health. Enzyme inhibition, particularly targeting *α*‐amylase and *α*‐glucosidase, represents a promising strategy for controlling blood glucose levels (Xu et al. 2018). *L. barbarum* polysaccharides primarily modulate glucose uptake (Cai et al. 2017). Phenolic compounds in *Lycium barbarum* , such as flavonoids and phenolic acids, have demonstrated notable hypoglycemic effects (Di et al. 2021). Phenolic extracts from new *L. barbarum* varieties exhibit *α*‐amylase inhibition rates ranging from 9.6% to 82.6% and *α*‐glucosidase inhibition rates between 5.7% and 15.3% (Wojdyło et al. 2018). Phenolic extracts from other plant species (Erythropalum scandens Blume (Adhikari et al. 2023) and *Artemisia dracunculus* (Güvenalp et al. 2017)) also demonstrate significant inhibitory effects on both *α*‐amylase and *α*‐glucosidase activities. At a concentration of 10 mg/mL, phenolic extracts and their components effectively delay sugar digestion and absorption, thereby reducing blood glucose levels. Even at low concentrations, the *α*‐amylase inhibition rate remains between 20% and 60% (Żołnierczyk et al. 2021). Also, the ethanol extract of *L. barbarum* phenolics has shown *α*‐glucosidase inhibition with an IC_50_ value of 129.46 μg/mL (Katanić et al. 2022).

However, the effects of different purification levels on the content, species distribution, and biological activities of phenolic compounds are still poorly understood. Therefore, this study aims to address this gap by systematically analyzing phenolic compounds at different purification levels in *L. barbarum* fruits from Ningxia. We evaluated the content, species, and enzyme inhibitory activity of phenolic extracts with varying purities to explore their performance at the biological activity level. Through this study, we intend to elucidate the potential relationships between content, species, and activity, thereby providing valuable insights and scientific support for the in‐depth study of *L. barbarum* phenolic compounds. Our findings emphasize the significance of advanced analytical techniques and purification methods in understanding and harnessing the bioactive potential of phenolic compounds.

## 2 Materials and Methods

### 2.1 Materials and Reagents

The raw material, *L. barbarum*, was obtained from Ningxia, China, and stored at room temperature in the laboratory conditions. The reagents and chemicals used in the study were obtained from the following suppliers: Folin–Ciocalteu phenol reagent (Shanghai Yuanye Biotechnology Co. Ltd.), sodium carbonate (Na_2_CO_3_), n‐hexane, petroleum ether, and methanol (analytical grade) (Tianjin Damao Chemical Reagent Factory), aluminum nitrate [Al(NO_3_)_3_] (Shanghai Shanpu Chemical Co. Ltd), hydrogen peroxide (H_2_O_2_) (Tianjin Guangfu Technology Development Co. Ltd), sodium hydroxide (NaOH) (Tianjin Beilian Fine Chemicals Development Co. Ltd), heavy water (Shanghai Baifei Chemical Technology Co. Ltd), *α*‐glucosidase and 4‐nitrophenyl‐*α*‐d‐glucopyranoside (Chengdu Aikeda Chemical Reagent Co. Ltd), *α*‐amylase (Beijing Solabo Technology Co. Ltd), and acarbose (Merck Life Sciences Co. Ltd). The experimental‐grade macroporous D101 resin was procured from Tianjin Guangfu Fine Chemical Research Institute, China, while Sephadex LH‐20 resin was obtained from Beijing Ruida Henghui Technology Development Co. Ltd. The instrumental equipment used in the study included the TU‐1810 UV–Vis spectrophotometer (Beijing Puxie General Instruments Co. Ltd), the iS50 FT‐IR Fourier transform infrared spectrometer (HP), an ultra‐performance liquid preparative chromatograph (Büchi, Switzerland), the Bruker Avance Neo 400 NMR spectrometer (Bruker Technology Co. Ltd), and the Orbitrap Exploris mass spectrometer (Thermo Fisher Scientific [China] Co. Ltd). For HPLC analysis, chromatographic‐grade formic acid (Sinopharm Chemical Reagent Co. Ltd., China) and acetonitrile (Fisher Chemical Company Limited, USA) were used.

### 2.2 Extraction, Separation, and Purification of Phenolic Compounds

C1 extract was obtained from *L. barbarum* via ethanol extraction and then subjected to macroporous D101 resin column chromatography for elution. The elution process employed ethanol solutions with volume fractions of 30%, 50%, 70%, and 95% (v/v), respectively. The eluted fractions were collected and designated as D1 (Fraction 1), D2 (Fraction 2), D3 (Fraction 3), and D4 (Fraction 4). A certain amount of D1 was subsequently subjected to Sephadex LH‐20 column chromatography to yield components S1 and S2. Component S1 was further purified via HPLC preparative chromatography to obtain P1. For the HPLC analysis, mobile phase A consisted of an aqueous solution with 0.1% formic acid, while mobile phase B consisted of a methanol solution with 1% formic acid. The flow rate was set at 18 mL/min, the column temperature was maintained at 25°C, and the detection wavelengths were 280 nm and 360 nm at the injection volume of 500 μL. The gradient elution conditions were as follows: (1) 0–11 min, linear gradient from 15% to 30% mobile phase A (i.e., mobile phase B decreases from 85% to 70%); (2) 11–15 min, linear gradient from 30% to 15% mobile phase A (i.e., mobile phase B increases from 70% to 85%); (3) 15–20 min, isocratic elution with 15% mobile phase A (85% mobile phase B). The purified part P1 was collected based on chromatographic peaks for subsequent structural identification (Zhou et al. 2016). In this experiment, target compounds C1, D1, S1, and P1, differing in purity, were selected for analytical purposes.

### 2.3 Determination of Total Phenols and Total Flavonoids

We used the colorimetric method described in a previous study (Yuan et al. 2022). We measured the absorbance at 765 nm using a UV–Vis spectrophotometer (TU‐1810, Beijing Purkinje GENERAL Instrument Co. Ltd.). The TPC was expressed as gallic acid equivalent (GAE) following the calibration curve with a linear range of 50–1000 μg/mL. The total flavonoids content (TFC) was determined using the colorimetric method described in a previous study (Islam et al. 2017). Absorbance was measured at 510 nm using a UV–Vis spectrophotometer (TU‐1810, Beijing Purkinje GENERAL Instrument Co. Ltd.). The TFC was expressed as catechin equivalent (CAE) based on a calibration curve with a linear range of 50–1000 μg/mL.

### 2.4 Comparative Analysis of the Number of Compounds Identified by UPLC‐MS in C1, D1, and S1

For comparative analysis, we accurately weighed 50 mg of lyophilized samples (C1, D1, and S1) and transferred them into 10 mL centrifuge tubes. Then after, 4 mL of an 80% methanol solution was added, followed by sonication at 5 kW for 80 min, then centrifugation at 8000 rpm for 10 min. The supernatant was collected and diluted to a final volume of 5 mL in a volumetric flask. The solution was then filtered through a 0.45 μm or 0.22 μm filter membrane for UPLC analysis. The Waters Acquity TQD system (Milford, MA, USA) was used to perform the UPLC analysis. At 30°C, the Waters Acquity BEH C18 column (100 × 2.1 mm, 1.7 μm) was kept at a constant flow rate of 0.3 mL/min for the elution process. 0.1% Formic acid in water (Phase A) and 0.1% formic acid in methanol (Phase B) made up the mobile phase. From 0 to 1 min, the elution gradient was 5% Phase B. From 1 to 24 min, it increased linearly to 100% Phase B. From 24.1 to 27.5 min, it maintained 100% Phase B. From 27.6 to 30 min, it returned to 5% Phase B. The system was injected with a 5 μL aliquot of the sample. Mass spectrometry (MS) analysis was conducted using an Orbitrap 1200 system (Ma, Wang, et al. 2023; Ma, Sun, et al. 2023). The instrument was operated in Data‐Dependent Acquisition (DDA) mode, with a primary full‐scan mass range of 50–1000 m/z. Key parameters included an auxiliary gas flow rate of 35, a sheath gas flow rate of 10, a purge gas flow rate of 1, and a spray voltage of 2.8 kV. The capillary temperature was maintained at 300°C, while the auxiliary gas heater was set to 280°C. For MS^2^ analysis, the resolution was configured to 17,500, with an AGC target of 5 × 10^6^, an isolation window of 0.4 m/z, and a dynamic exclusion time of 4.5 s. The s‐lens RF level was set to 50 V, and in negative ion mode, the maximum injection time was 50 ms with an AGC target of 3.0 × 10^6^. Data acquisition was performed using Xcalibur 4.0 software, and subsequent data preprocessing and analysis were conducted using the Compound Discover Data Preprocessing Platform. This methodology ensured the generation of accurate and reliable data to support subsequent research.

### 2.5 Study on Hypoglycemic Enzyme Activity of Phenolic Extracts With Different Purity

The inhibitory effect of phenolic extracts from *L. barbarum* with varying purities (C1, D1, and S1) on *α*‐amylase activity was investigated. Extracts were prepared at concentrations of 0.4, 0.8, 1.2, 1.6, 2.0, 4.0, 6.0, 8.0, and 10.0 mg/mL. Similarly, *α*‐amylase solutions were prepared at the same concentrations (Abdelli et al. 2021; Figueiredo‐González et al. 2016; Xin et al. 2018). We measured the absorbance at 540 nm using a UV–Vis spectrophotometer with acarbose as the positive control. For the *α*‐amylase inhibition rate, we used the following (1–1) formula:

$$\text{Inhibition rate}\;\left(\%\right)=\left(A-B\right)/\left(C-D\right)\times 100\;\left(1–1\right)$$

where *A* is the absorbance of the inhibitor group, *B* is the absorbance of the buffer (background control), *C* is the absorbance of the buffer without inhibitor (blank group), and *D* is the absorbance of the buffer without both enzyme and inhibitor (blank control).

The inhibitory effect of phenolic extracts on *α*‐glucosidase activity was studied using the same. Extracts were prepared at concentrations of 0.4, 0.8, 1.2, 1.6, 2.0, 4.0, 6.0, 8.0, and 10.0 mg/mL, while *α*‐glucosidase solutions were prepared at 2.0, 4.0, 6.0, 8.0, and 10.0 mg/mL. Absorbance (OD) was measured every minute at 405 nm using a UV–Vis spectrophotometer (De Oliveira Raphaelli et al. 2019; Magana‐Barajas et al. 2021). Acarbose served as the positive control, and three parallel samples were analyzed for each group. The *α*‐glucosidase inhibition rate was calculated using the following (1–2) formula:

$$\text{Inhibition rate}\;\left(\%\right)=\left(A-B\right)/\left(C-D\right)\times 100\;\left(1–2\right)$$

where *A* is the absorbance of the inhibitor group, *B* is the absorbance of the buffer (background control), *C* is the absorbance of the buffer without inhibitor (blank group), and *D* is the absorbance of the buffer without both enzyme and inhibitor (blank control).

### 2.6 UV–Vis Spectral Analysis of P1

Phenolic extracts of varying purities (20 mg each) were dissolved in 10 mL of methanol as the solvent. Methanol was used as the blank solution in this analysis. The UV–Vis spectrum of the P1 sample was measured using a UV–Vis spectrophotometer (Agilent Technologies, China) within the wavelength range of 200–700 nm, and a complete spectral scan was recorded.

### 2.7 FTIR Analysis of P1

We weighed 5–10 g of *L. barbarum* sample, froze it in liquid nitrogen, ground it into powder, bagged it, sealed it, labeled it, and placed it in a dryer before testing (Souza et al. 2012). Then, potassium bromide was dried in an electric blast drying oven at 100°C for about 4 h and kept in a dryer for later use. Using the KBr tableting method, 1 mg of sample was weighed, potassium bromide was mixed, and the resulting mixture was ground at 1:150 (mass ratio). After the tablet machine was made into transparent or translucent ingots, it was placed in the sample pool for testing. A total of 32 scans were made at 4000 cm^−1^–400 cm^−1^, and the interference of water and carbon dioxide was deducted during scanning. The original data source was obtained by collecting sample information using OMNIC 7.3 intelligent software, and the correlation analysis was conducted.

### 2.8 NMR Analysis of P1

A 100 mg sample of P1 was dissolved in DMSO‐d6 reagent and allowed to equilibrate at room temperature for 3 h. ^1^H NMR spectroscopy was conducted on a Bruker Avance^−1^ spectrometer (Karlsruhe, Germany), following the previously reported method (Onyancha et al. 2023). The phenolic monomer was dissolved in a heavy water mixture for ^1^H NMR analysis, with the spectrometer operating at a temperature of 300 K.

### 2.9 UPLC‐MS Analysis of P1

The UPLC‐MS analysis for P1 was conducted using the same methodology outlined in Section 2.4, ensuring consistency in the analytical approach.

### 2.10 Statistical Analysis

This study used the SPSS 22.0 and 26 software for effective statistical comparisons and evaluations. One‐way ANOVA or Duncan's multiple range test was employed for data analysis, with results considered statistically significant at *p* < 0.05. The EC_5_ values for hypoglycemic enzyme determinations were calculated using the linear regression equation provided by SPSS 22.0 software. Graphical representations were created using Origin Pro 2023 sr0 software, and the overall data analysis was performed with SPSS 26.0 software to ensure the scientific accuracy and reliability of the results, thereby providing robust data support for the research conclusions.

## 3 Results

### 3.1 Yield Analysis of Phenolic Extracts With Different Purity

**FIGURE 1:**
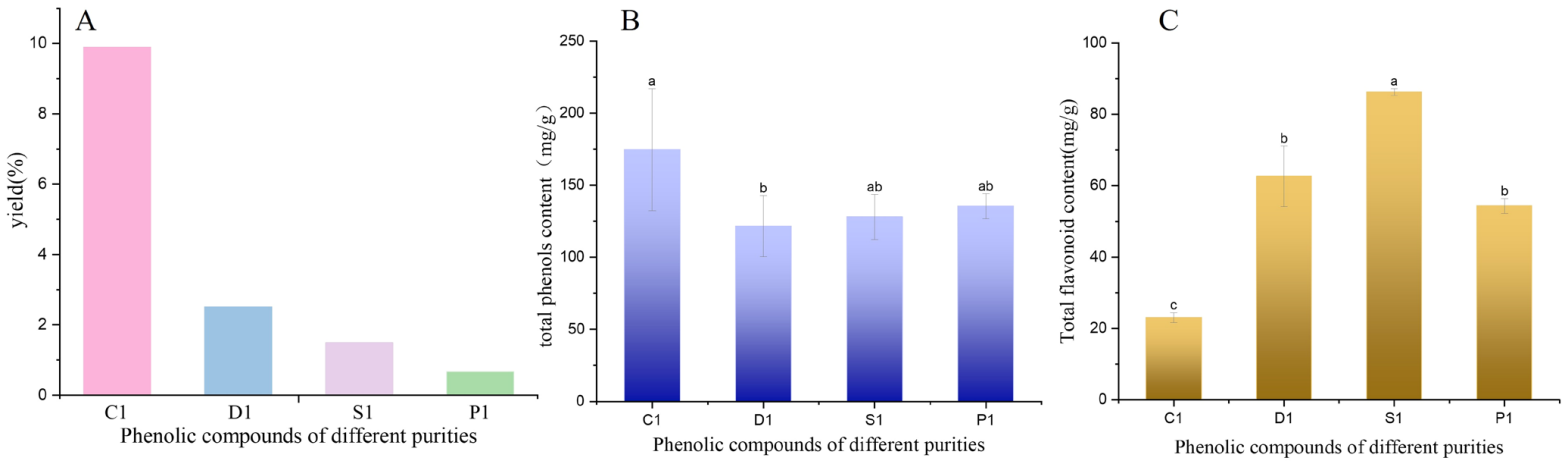
Yield of phenolic compounds of *L. barbarum* with respect to different purification methods (A, B) total phenolic and (C) flavonoid content of differently purified Ningxia *L. barbarum* phenolic compounds [Bars with different letters are significantly different (*p* < 0.05)].

During the extraction and separation process, different separation methods gradually remove impurities for selective enrichment of target phenolic compounds. It can be clearly seen from Figure 1A that the yield of phenolic extracts of different purity *L. barbarum* was in the range of 1%–10%. Low‐concentration ethanol (30%) extraction preferentially eluted polar impurities and some phenolic compounds. With the increase of ethanol concentration, the purity of the eluted phenolic compounds increased, but with the removal of many impurities, the yield of the obtained fraction (D1) decreased. This was because the crude extract C1 had many impurities, while D1 was the preliminary product with fewer impurities, resulting in a lower yield. The Sephadex LH‐20 chromatography column used the molecular sieve effect (Lucia et al. 2020) to further separate the compounds in D1 based on molecular size and shape, and removing impurities that did not match the molecular weight or had different affinity with the gel, which further improved the purity of S1, but also reduced the amount of product, resulting in a lower yield. HPLC used precise elution conditions to finely separate the target phenolic compounds in S1 from remaining impurities. Although high‐purity P1 was obtained, many impurities were removed, resulting in an extremely low yield of P1. From a theoretical perspective, this may be because during the purification process, increased solute diffusion power and lowered solution viscosity (Hao et al. 2020) enhance solute transfer from the raw material to the extract. With the more refined purification process, the purity of phenolic compounds improves, but the yield is compensated due to the removal of impurities.

### 3.2 Content Analysis of Total Phenols and Total Flavonoids in Phenolic Extracts With Different Purity

Through the determination of total phenol content of *L. barbarum* in Ningxia, the change in total phenol content of phenolic extracts of different purity *L. barbarum* was analyzed, and then the difference in total phenol content between different purity compounds was clarified. Figure 1B shows that the total phenol content of *L. barbarum* phenolic compounds at different purity levels exhibited significant differences. The total phenol content of crude extract C1 was the highest (199.986 mg/g), and the total phenol content of D1 after elution by macroporus D101 resin column was the lowest (121.588 mg/g) with the advance of purification steps. However, the total phenol content of S1 and P1 obtained by HPLC after separation by Sephadex LH‐20 column was between C1 and D1, and there was no significant difference in the total phenol content of S1 and P1. Figure 1C shows that the total flavonoid content varied among the extracts. The total flavonoid content of S1 was the highest (86.247 mg/g), and the total flavonoid content of crude extract C1 was the lowest (23.007 mg/g).

The change trends of total phenols and total flavonoids in different purity extracts were significantly different. The crude extract C1 had high total phenols and low total flavonoids. After purification of S1, the total flavonoids increased significantly, while the total phenols remained at a medium level. This shows that the content of total phenols and total flavonoids did not change synchronously during the purification process, and each followed different laws. This difference stemmed from the different selectivity of different methods during the separation and purification process (Wen et al. 2017). In the elution stage of macroporus D101 resin column, different volume fractions of ethanol solutions had different elution capabilities for different compounds. While the total phenols were initially enriched, some flavonoids were also taken away, resulting in low content of total phenols and total flavonoids in D1. However, the separation and enrichment of flavonoids by Sephadex LH‐20 chromatography column was more effective, resulting in a significant increase in the total flavonoid content of S1. The total phenols in S1 have been lost to a certain extent in the earlier purification step, resulting in a high total flavonoid content and relatively medium total phenol content in S1. In addition, the different chemical structures and properties of total phenols and total flavonoids (Wang et al. 2022) influenced their adsorption on the column packing, inner wall of the pipeline, and the losses during sample transfer and eluent collection, which further increased the difference in their content in different purity extracts. Our data revealed the selective influence of purification process on the total phenol and flavonoid content which is crucial for optimization of extraction to obtain desired profiles for various application.

### 3.3 Composition Difference Analysis of Phenolic Substances With Different Purity Based on UPLC‐MS

UPLC‐MS analysis of different *L. barbarum* phenolic extracts revealed a total of 78 phenolic substances, including 16 saponins, 23 flavonoids, 15 phenolic acids, 6 phenylpropanes, and 18 phenolic amides as shown in Table 1. The crude phenolic extracts of *L. barbarum* and the compounds isolated and purified by D 101 macroporous resin showed that the number of flavonoids and phenolic acids in the latter was significantly reduced. The Sephadex LH‐20 glucan gel assisted purification can realize the purification of phenolic acids. During the purification process from D 101 macroporous resin to Sephadex LH‐20 glucan gel, the phenolic acids in the phenolic extracts of *L. barbarum* were reduced.

**TABLE 1:** Mass spectrometry information of phenolic compounds in *Lycium barbarum* under positive and negative ion mode (ESI) by high‐resolution mass spectrometry.

Category	Serial number	RT	Ion mode	Mother ion, daughter ion	C1 compound	D1 compound	S1 compound	Molecular formula	PPM	References
Saponins	1	20.057	[M + H]^+^	487.3579/163.0399	P‐coumaric acid 4‐O‐dihexanoside	P‐coumaric acid 4‐O‐dihexanoside	—	C_21_H_27_O_13_	0.39	Qian et al. (2020)
	2	0.731	[M − H]¯	649.2316/487.1436/325.0916/163.0399	P‐coumaric acid 4‐O‐trihexanoside	—	—	C_27_H_38_O_18_	1.16	Qian et al. (2020)
	3	21.264	[M + H]^+^	487.3579/323.0916/163.0399	P‐coumaric acid 4‐O‐dihexanoside	P‐coumaric acid 4‐O‐dihexanoside	—	C_21_H_27_O_13_	2.37	Qian et al. (2020)
	4	0.801	[M − H]¯	649.2316/487.1436/163.0399	P‐coumaric acid 4‐O‐trihexanoside	—	—	C_27_H_38_O_18_	0.51	Qian et al. (2020)
	5	0.852	[M + H]^+^	796.3457/634.2982/472.2484/334.1785/191.0547	Di (dihydrocaffeoyl) arginine‐dihexoside	—	—	C_37_H_55_N_3_O_16_	1.85	Qian et al. (2020)
	6	0.747	[M − H]¯	632.2831/470.2300/334.1785/191.0547	Caffeoyl (dihydrocaffeoyl) arginine dihexoside	Caffeoyl (dihydrocaffeoyl) arginine dihexoside	Caffeoyl (dihydrocaffeoyl) arginine dihexoside	C_37_H_53_N_3_O_16_	1.54	Qian et al. (2020)
	7	28.815	[M − H]¯	325.1843/298.8872/163.0398/145.0276/119.0497	P‐coumaric acid‐O‐glycoside	P‐coumaric acid‐O‐glycoside	P‐coumaric acid‐O‐glycoside	C_15_H_16_O_8_	0.02	Lv et al. (2017)
	8	28.817	[M + H]^+^	447.8751/301.2/179.4	Quercitrin	Quercitrin	—	C_21_H_20_O_11_	3.84	Zhou (2017)
	9	21.754	[M + H]^+^	463.2652/316.3/179.4	Myricetrin	—	—	C_21_H_20_O_12_	1.19	Zhou (2017)
	10	21.031	[M − H]¯	355.2278/191.0	Scopolin	Scopolin	Scopolin	C_17_H_13_NO_4_	3.28	Lv et al. (2017)
	11	20.811	[M − H]¯	369.2434	Fraxinin	Fraxinin	—	C_16_H_18_O_10_	0.21	Lv et al. (2017)
	12	20.403	[M + H]^+^	463.3007	Myristin	—	—	C_17_H_23_O_9_	1.32	Lv et al. (2017)
	13	7.794	[M − H]¯	853.5761	N‐caffeoyl‐N′‐dihydrocaffeoyl spermidine‐diglucoside	—	—	C_40_H_61_N_4_O_16_	3.89	Qian et al. (2023)
	14	28.838	[M + H]^+^	855.7698	N‐N′‐di‐(dihydrocaffeoyl) arginine di‐glucoside	—	—	C_40_H_61_N_4_O_16_	0	Qian et al. (2023)
	15	0.852	[M + H]^+^	796.3457	N‐caffeoyl‐N′‐dihydrocaffeoyl spermidine‐diglucoside	N‐caffeoyl‐N′‐dihydrocaffeoyl spermidine‐diglucoside	N‐caffeoyl‐N′‐dihydrocaffeoyl spermidine‐diglucoside	C_37_H_54_N_3_O_16_	1.85	Qian et al. (2023)
	16	0.885	[M + H]^+^	634.2937	N‐caffeoyl‐N′‐dihydrocaffeoyl spermidine‐monoglucoside	N‐caffeoyl‐N′‐dihydrocaffeoyl spermidine‐monoglucoside	N‐caffeoyl‐N′‐dihydrocaffeoyl spermidine‐monoglucoside	C_31_H_44_N_3_O_11_	1.31	Qian et al. (2023)
Flavonoid	17	22.306	[M − H]¯	339.1998	Aesculin	Aesculin	Aesculin	C_15_H_16_O_9_	3.5	Lv et al. (2017)
	18	28.838	[M − H]¯	367.9184	Curcumin	Curcumin	Curcumin	C_21_H_20_O_6_	3.2	Lv et al. (2017)
	19	0.818	[M − H]¯	191.0197	Scopoletin	Scopoletin	Scopoletin	C_10_H_8_O_4_	0.14	Lv et al. (2017)
	20	28.84	[M + H]^+^	291.8882/182.0579	Catechol	—	—	C_15_H_15_O_6_	0.56	Li et al. (2019)
	21	14.21	[M − H]¯	286.2387/217.2/199.2	Kaempferol	Kaempferol	Kaempferol	C_15_H_10_O_6_	0.01	Zhou (2017)
	22	28.826	[M − H]¯	316.9211	Isorhamnetin	Isorhamnetin	Isorhamnetin	C_16_H_12_O_7_	4.16	Zhou (2017)
	23	28.28	[M − H]¯	610.1812	Rutin	Rutin	Rutin	C_27_H_30_O_16_	1.58	Zhou (2017)
	24	22.291	[M + H]^+^	301.2100/179.2/151.1	Quercetin	—	—	C_15_H_10_O_7_	1.6	Zhou (2017)
	25	28.85	[M + H]^+^	269.8653/117.1/151.1	Apigenin	—	—	C_15_H_10_O_8_	2.71	Li et al. (2019)
	26	7.401	[M + H]^+^	331.2527/195.0293	3′,4′‐O‐dimethylquercetin	—	—	C17H15O7	1.86	Yang et al. (2015)
	27	21.183	[M + H]^+^	510.4852	(±)‐(7″R*, 8″R*)‐canabisine H	—	—	C_28_H_31_NO_8_	1.46	Lv et al. (2017)
	28	29.286	[M − H]¯	193.8156	Ethyl trans‐p‐coumarate	—	—	C_11_H_12_O_3_	1.9	Lv et al. (2017)
	29	21.785	[M − H]¯	223.0283	Ethyl trans‐ferulate	—	—	C_12_H_14_O_4_	4.9	Lv et al. (2017)
	30	18.282	[M − H]¯	233.1548	Syringenin	—	—	C_11_H_14_O_4_	0.04	Lv et al. (2017)
	31	0.876	[M − H]¯	202.1048	Trans‐coniferol	Trans‐coniferol	Trans‐coniferol	C_10_H_12_O_3_	0.11	Lv et al. (2017)
	32	28.791	[M + H]^+^	225.1335	Ethyl‐dihydroferulate	—	—	C_12_H_16_O_4_	1.37	Lv et al. (2017)
	33	8.359	[M + H]^+^	509.8829	Fabiatrin	—	—	C_21_H_26_O_13_	1.46	Lv et al. (2017)
	34	10.282	[M + H]^+^	377.1938/731.1	Scopolin	Scopolin	Scopolin	C_16_H_18_O_9_	1.14	Lv et al. (2017)
	35	19.84	[M + H]^+^	214.2155	Scopoletin	Scopoletin	Scopoletin	C_10_H_8_O_4_	4.81	Lv et al. (2017)
	36	28.856	[M‐H]¯	359.863	Pinoresinol	—	—	C_20_H_22_O_6_	0.36	Lv et al. (2017)
	37	21.821	[M + H]^+^	411.3074	Medioresinol	—	—	C_21_H_24_O_7_	1.81	Lv et al. (2017)
	38	8.115	[M + H]^+^	472.3/310.3	Cyanidin‐3‐O‐galactoside	—	—	C_21_H_21_O_11_	0.02	Li et al. (2019)
	39	28.82	[M + H]^+^	787.7842/625.1754/479.1177	Petunidin‐3‐O‐rutinoside‐5‐O‐glucoside	—	—	C_34_H_43_O_21_	0.29	Lucia et al. (2020)
Phenolic acids	40	21.326	[M − H]¯	163.1125	P‐coumaric acid	P‐coumaric acid	P‐coumaric acid	C_9_H_8_O_3_	1.49	Zhou (2017)
	41	23.832	[M + H]^+^	178.9589	Caffeic acid	—	—	C_9_H_8_O_4_	0.06	Zhou (2017)
	42	29.286	[M − H]¯	193.8156	Ferulic acid	—	—	C_10_H_10_O_4_	0	Zhou (2017)
	43	20.431	[M − H]¯	353.2121/731.1/377.3	3‐caffeoyl quinic acid (chlorogenic acid)	3‐caffeoyl quinic acid (chlorogenic acid)	—	C_16_H_18_O_9_	0.17	Lv et al. (2017)
	44	21.592	[M − H]¯	515.3200/353.0750/191.0547	Dicaffeoquinic acid	Dicaffeoquinic acid	Dicaffeoquinic acid	C_25_H_24_O_12_	0.03	Qian et al. (2020)
	45	29.286	[M − H]¯	193.8156	Trans‐ferulic acid	—	—	C_10_H_10_O_4_	1.9	Li et al. (2019)
	46	28.801	[M − H]¯	303.8866	Arachidonic acid	—	—	C_20_H_32_O_2_	4.69	Lv et al. (2017)
	47	21.785	[M − H]¯	223.0283	Sinapic acid	Sinapic acid	Sinapic acid	C_11_H_12_O_5_	0.05	Lv et al. (2017)
	48	22.291	[M + H]^+^	301.21	Gallogen	—	—	C_14_H_6_O_8_	2.77	Lv et al. (2017)
	49	7.794	[M − H]¯	168.8869	Vanillic acid	—	—	C_8_H_8_O_4_	0.05	Lv et al. (2017)
	50	0.777	[M + H]^+^	337.1699/175.0621	5‐O‐cafeoyl shikimic acid	5‐O‐cafeoyl shikimic acid	5‐O‐cafeoyl shikimic acid	C_16_H_17_O_8_	1.76	Yang et al. (2015)
	51	28.865	[M + H]^+^	247.1157/223.0	Trans‐sinapinic acid	—	—	C_11_H_2_O_5_	3.32	Lv et al. (2017)
	52	29.29	[M^+^ H]^+^	149.9521	Cinnamic acid	—	—	C_9_H_8_O_2_	0.95	Lv et al. (2017)
	53	29.85	[M + H]^+^	216.9217/192.9	Cis‐ferulic acid	Cis‐ferulic acid	Cis‐ferulic acid	C_10_H_10_O_4_	0.46	Lv et al. (2017)
	54	28.77	[M − H]¯	186.9057/163.0	Cis‐p‐coumaric acid	—	—	C_9_H_8_O_3_	3.82	Lv et al. (2017)
Phenylpropanoids	55	28.823	[M − H]¯	541.8168	Lycibarbarphenylpropanoid D	Lycibarbarphenylpropanoid D	—	C_22_H_30_O_14_Na	1.57	Lv et al. (2017)
	56	28.826	[M − H]¯	371.8613	Lycibarbarphenylpropanoid I	Lycibarbarphenylpropanoid I	Lycibarbarphenylpropanoid I	C_17_H_23_O_9_	0	Lv et al. (2017)
	57	28.823	[M − H]¯	541.8168	Lycibarbarphenylpropanoid C	Lycibarbarphenylpropanoid C	—	C_22_H_30_O_14_Na	0.13	Lv et al. (2017)
	58	28.777	[M − H]¯	645.8367	Lyciumamide P	—	—	C_36_H_36_N_2_O_8_	0.13	Lv et al. (2017)
	59	28.883	[M − H]¯	546.8354	Lyciumamide Q	—	—	C_36_H_36_N_2_O_8_	0.16	Lv et al. (2017)
	60	0.818	[M − H]¯	632.2047	Lycibarbarspermidine N	Lycibarbarspermidine N	Lycibarbarspermidine N	C_18_H_20_NO_5_	0.71	Lv et al. (2017)
Phenolamine amides	61	0.885	[M^+^ H]^+^	634.2937	N,N‐dihydrocaffeoyl, caffeoyl‐spermidine, hexasaccharide	N,N‐dihydrocaffeoyl, caffeoyl‐spermidine, hexasaccharide	N,N‐dihydrocaffeoyl, caffeoyl‐spermidine, hexasaccharide	C_25_H_31_N_3_O_4_	0.36	Qian et al. (2023)
	62	0.786	[M − H]¯	498.1944	N‐(4‐O‐β‐D‐glucopyranosyl‐cis‐feruloyl)‐tyramine	—	—	C_24_H_29_NO_9_	3.05	Lv et al. (2017)
	63	6.14	[M + H]^+^	498.3987	N‐cis‐feruloyl‐4‐O‐β‐D‐glucopyranosyl‐tyramine	—	—	C_24_H_29_NO_9_	1.75	Lv et al. (2017)
	64	28.845	[M − H]¯	282.9408	N‐cis‐p‐coumaroyl‐tyramine	N‐cis‐p‐coumaroyl‐tyramine	—	C_17_H_17_NO_3_	0.11	Lv et al. (2017)
	65	18.354	[M + H]^+^	314.3403	N‐cis‐feruloyl‐tyramine	—	—	C_18_H_19_NO_4_	4.84	Lv et al. (2017)
	66	21.649	[M + H]^+^	589.4257	N‐trans‐p‐coumaroyl‐tyramine	—	—	C_17_H_17_NO_3_	1.48	Lv et al. (2017)
	67	0.788	[M − H]¯	307.1147	Feruloyl‐citamide	—	—	C_14_H_20_N_2_O_3_	0.29	Li et al. (2019)
	68	29.955	[M − H]¯	235.926	Coumaryl‐putrescine	—	—	C_17_H_17_NO_4_	3	Li et al. (2019)
	69	17.231	[M − H]¯	265.1479	Feruloyl‐putrescine	Feruloyl‐putrescine	Feruloyl‐putrescine	C_14_H_20_N_2_O_3_	0.17	Li et al. (2019)
	70	0.784	[M + H]^+^	219.0254	N‐cinnamyl‐putrescine	N‐cinnamyl‐putrescine	—	C_9_H_9_NO	0.75	Li et al. (2019)
	71	15.371	[M + H]^+^	302.2674	Dihydro‐n‐caffeoyl tyramine	—	—	C_17_H_20_NO_4_	3.89	Li et al. (2019)
	72	15.718	[M + H]^+^	330.2622	Trans‐N‐feruloyldopamine	—	—	C_18_H_20_NO_5_	3.11	Li et al. (2019)
	73	21.084	[M + H]^+^	300.2883	Trans‐n‐caffeoyl tyramine	—	—	C_17_H_18_NO_4_	4.54	Li et al. (2019)
	74	21.084	[M + H]^+^	300.2883/163.0384/137.0855	N‐caffeoyl tyramine	—	—	C_17_H_18_NO_4_	4.54	Yang et al. (2015)
	75	0.789	[M + H]^+^	284.1511/147.0455/137.0851	N‐coumaryl tyramine	—	—	C_17_H_18_NO_3_	0.49	Yang et al. (2015)
	76	18.354	[M + H]^+^	314.3403/177.0563/137.0853	N‐feruloyl tyramine	—	—	C_18_H_20_NO_4_	4.84	Yang et al. (2015)
	77	17.704	[M + H]^+^	316.3193/177.0559/153.0779	N‐caffeyldopamine	N‐caffeyldopamine	N‐caffeyldopamine	C_17_H_18_NO_5_	3.32	Yang et al. (2015)
	78	20.908	[M + H]^+^	344.3141/163.0387/167.0958	N‐feruloyl‐3‐O‐methyldopamine	—	—	C_19_H_22_NO_5_	2.61	Yang et al. (2015)

**FIGURE 2:**
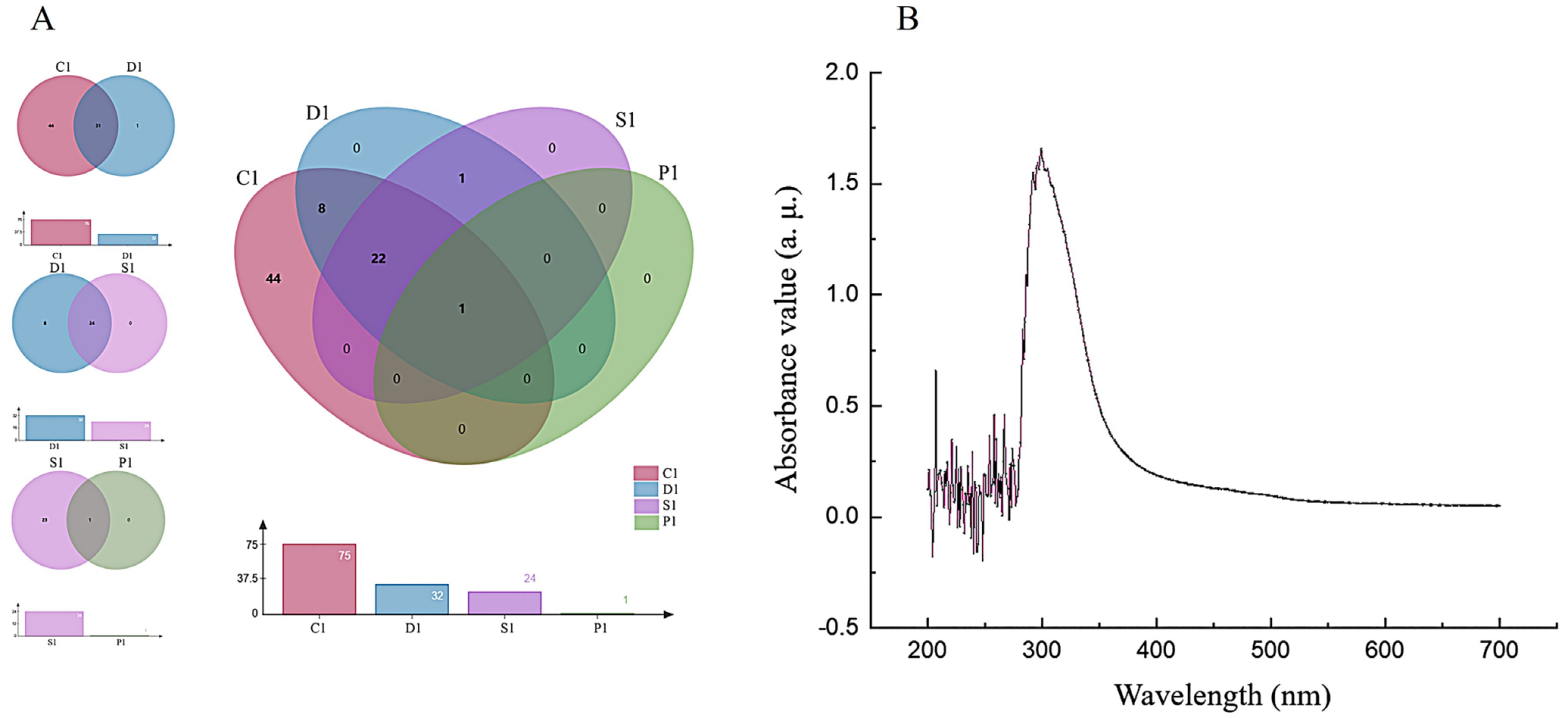
Veen diagram of phenolic extracts from *L. barbarum* with different purification methods (A); P1 full wavelength UV scan of *L. barbarum* phenolic dextran gel extract (B).

Table 1 and Figure 2A show that the types of compounds decreased significantly during the process from C1 to D1. In the saponins, some compounds such as “P‐coumaric acid 4‐O‐trihexanoside” did not appear in D1; the number of flavonoids also decreased, such as “myricetrin” was missing in D1; phenolic acids were also decreased, and “caffeic acid” no longer existed in D1. This is mainly because during the elution process of the macroporus D101 resin column, compounds with inappropriate polarity or weak adsorption were either eluted or not retained according to the polarity of the compound and the difference in adsorption capacity with the resin (Yang et al. 2024), reducing the total number of phenolic substances. The total phenolic content of C1 was the highest in crude extract, and the total phenolic content decreased with the decrease of compound species, such as the removal of some phenolic compounds during the process from C1 to D1. This shows that in the early stage of purification, the removed compounds contained more phenolic substances, resulting in a decrease in total phenolic content, which was the same as the results reported in the literature (Chu et al. 2018). During the purification process from C1 to D1, although the flavonoid content decreased, the total flavonoid level remained relatively stable. This was likely due to the removal of other interfering impurities, which compensated for the loss of flavonoids. From D1 to S1, both the diversity and content of saponins were markedly reduced; for instance, “Di (dihydrocaffeoyl) arginine‐dihexoside” was no longer detected. Phenolic acids were further eliminated, and “Dicaffeoylquinic acid” also disappeared. In contrast, flavonoids and phenylpropanoids remained relatively stable. This is due to the molecular sieve action of the Sephadex LH‐20 chromatography column, which screened compounds based on molecular sizes, so that some compounds in the saponins and phenolic acids that did not match the molecular size could be isolated (Zhang et al. 2022), while the separation effect of flavonoids and phenylpropanes was relatively weak. From D1 to S1, the total phenol content changed relatively little, probably because other non‐phenols such as proteins or phenolic compounds that had little effect on the total phenol content were removed at this stage. From D1 to S1, due to the further removal of phenolic acids and saponins, the proportion of flavonoids was relatively increased, resulting in an increase in the total flavonoid content of S1.

### 3.4 UV–Vis Spectral Analysis of P1

Figure 2B shows that the maximum absorption wavelength of phenols was around 320 nm, and that of flavonoid impurities existed around 280 nm and 320 nm. (Taniguchi et al. 2023). It can also be seen from the figure that before the phenolic extract of *L. barbarum* was extracted by n‐hexane and petroleum ether, the absorption peak of flavonoid impurities was significantly higher than that of phenolic acids; and after extraction, the characteristic peak of flavonoids in the extract was significantly higher than that of flavonoid impurities, which indicated that petroleum ether and n‐hexane can effectively remove a certain amount of flavonoid impurities in the phenolic extract. After purification by D 101 macroporous resin, many flavonoids, phenolamides, saponins, phenolic acids, and phenylpropanes were detected in fraction 1; after purification by Sephadex LH‐20 dextran gel, the remaining flavonoids and phenylpropanes were concentrated and lyophilized. The wavelength of the phenolic extracts of different purity *L. barbarum* obtained after extraction and two separations and purification ranged between 280 and 360 nm. This spectral analysis provides an important basis for in‐depth understanding of the changes in substances during the extraction and purification process and helps to further optimize the extraction and purification process.

### 3.5 FTIR Analysis of P1

**FIGURE 3:**
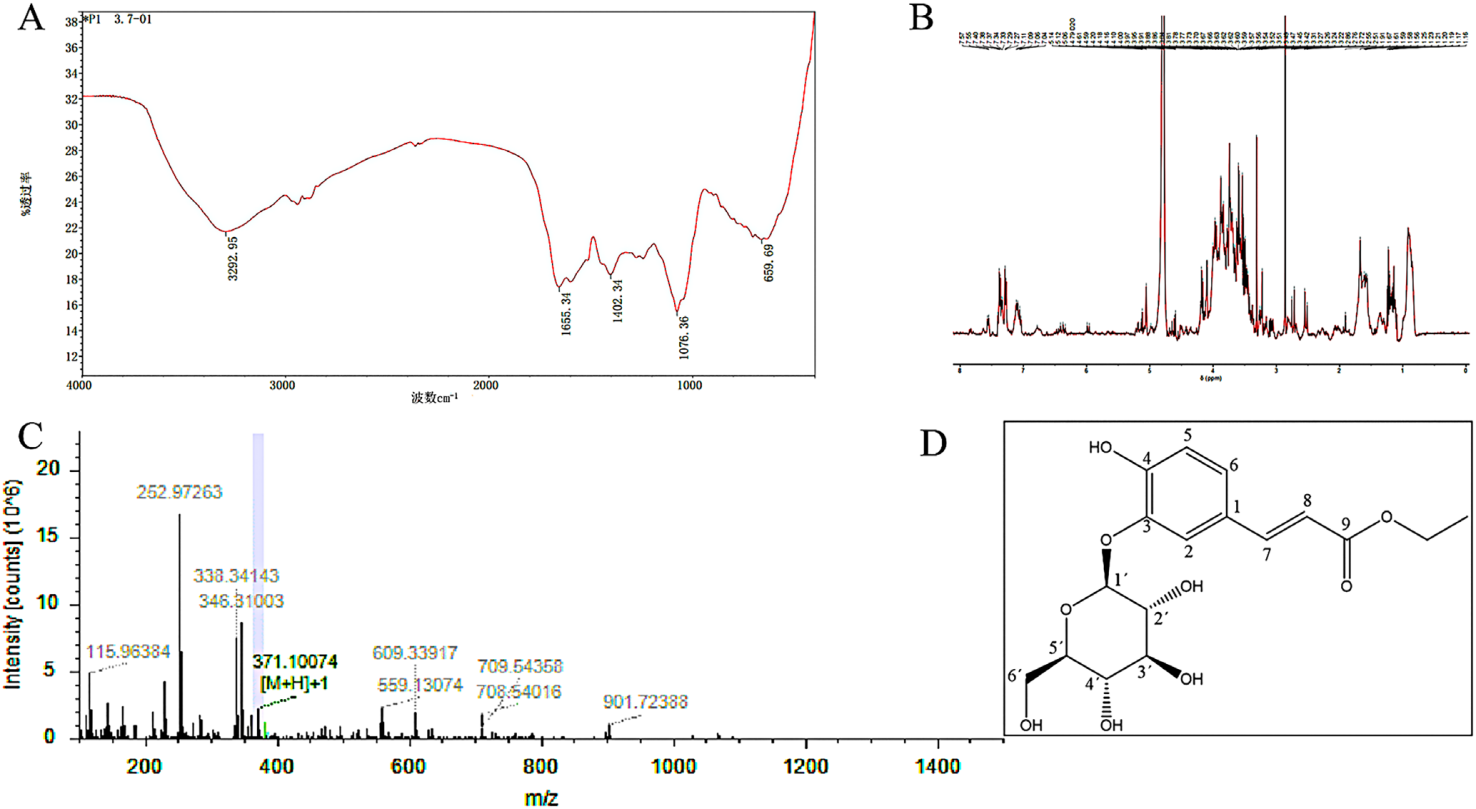
(A) FTIR of P1, (B) mass spectrum of P1, (C) P1 ^1^H NMR, (D) plane structure diagram of P1.

As can be seen from Figure 3A, the *L. barbarum* phenolic extract monomers prepared by high‐performance liquid chromatography exhibited strong different degrees of infrared absorption at 3292, 2925, 2300, 1655, 1402, 1076, and 659 cm^−1^. The absorbance at the same wavenumber varied, with higher absorbance indicating higher content and lower absorbance value indicating lower content. Among them, the stretching vibration of OH was about 3292 cm^−1^, and the stretching vibration of alkanes CH was near 2925 cm^−1^. 1655 cm^−1^ was the stretching vibration of C=O vibration, 1402 cm^−1^ was the in‐plane bending vibration of phenolic hydroxyl and alcoholic hydroxyl, 1076 cm^−1^ was the stretching vibration of the in‐plane bending of substituted benzene CH, and at 659 cm^−1^ was the out‐of‐plane bending vibration of alcoholic hydroxyl (Wu et al. 2022), where the vibration characteristics of CH and phenolic hydroxyl were similar to those of phenylpropanol and phenamide in *L. barbarum*. Based on the spectral characteristics, it is inferred that the phenolic extract P1 of *L. barbarum* may contain phenylpropanol, phenolamide, and other chemical components. To summarize, these infrared adsorption spectra revealed the presence of different functional groups and chemical components, revealing the composition and structures of these compounds.

### 3.6 NMR Analysis of P1

Lycibarbarphenylpropanoid I: pale yellow amorphous powder; ESIMS (positive) m/z 371.1007 [M + H]^+^; ^1^H NMR data are shown in table 2 (Katanić et al. 2022; Zhou 2017). It is speculated that the obtained *L. barbarum* phenolic extract P1 was Lycibarbarphenylpropanoid I, and its chemical structure is shown in Figure 3B P1. The molecular formula of the compound was determined to be C_17_H_22_O_9_ (7 degrees unsaturated). A detailed analysis of its ^1^H NMR data (Table 2) inferred the planar structure as shown in Figure 3C,D for glycosides, with ethyl caffeate as glycoside. Comparison of the ^13^C NMR data of 13 sugar units with the glycosides recorded previously (Qian et al. 2023) indicated that the glycochain of 9 was glucopyranose *β*‐d‐glucopyranose. The glucopyranosyl group was identified as *β*‐d‐Glc based on the coupling constant of isomeric proton and acid hydrolysis. Therefore, Compound 9 was identified as 3‐O‐*β*‐D‐glucopyranosylcaffeate ethyl ester and named Lycibarbarphenylpropanoid I.

**TABLE 2:** ^1^H NMR (400 MHz) data of the P1.

Number	*δ*_H_ (*J*, Hz)
2	7.34, *d* (6.4)
5	7.28, *d* (7.3)
6	7.37, *d* (7.3)
7	7.56, *d* (8.2)
8	6.39, *d* (16.0)
1′	4.60, *d* (7.9)
9‐OCH_2_CH_3_	4.19, *q* (7.2)
9‐OCH_2_CH_3_	1.23, *t* (7.2)

### 3.7 Comparison of Hypoglycemic Enzyme Activities of Different Purity Phenolic Extracts

**FIGURE 4:**
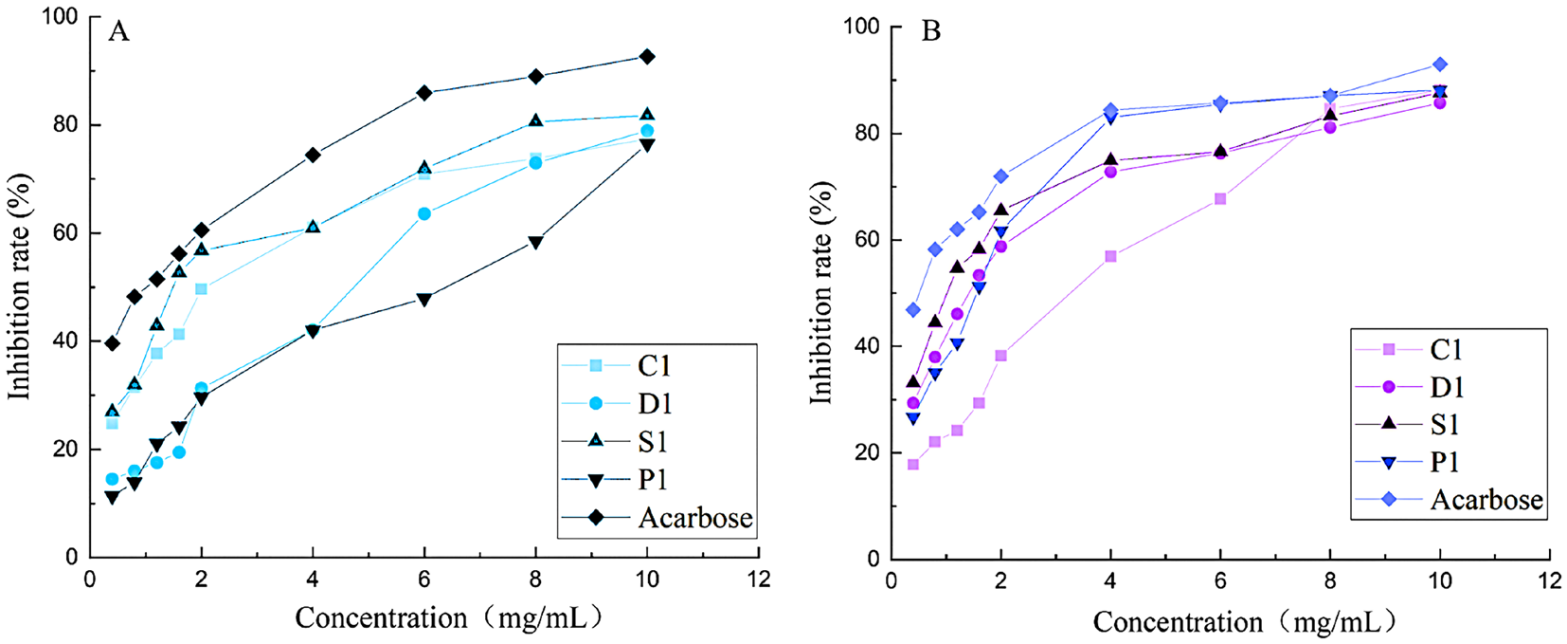
Inhibition rate of *α*‐amylase activity (A) and *α*‐glucosidase activity (B) by phenolic compounds obtained from different purification methods.

As shown in Figure 4, phenolic substances of different purity inhibited the activity of *α*‐amylase. Among them, C1 showed the highest inhibitory rate of *α*‐amylase activity at 77.41%, with a semi‐inhibitory concentration of 0.325 mg/mL; phenolic substances of different purity also inhibited the activity of *α*‐glucosidase. S1 showed the highest inhibitory rate of *α*‐glucosidase activity at 85.17%, with a semi‐inhibitory concentration of 0.065 mg/mL. Based on the above research results, phenolic extracts of different purity had a certain degree of hypoglycemic ability. Further analysis found that the hypoglycemic activity was closely related to the types and content of compounds present in the extracts. Through the analysis of the content and variety changes of different purity compounds, during the process from C1 to D1, 14 flavonoids were reduced, and 13 phenolamides were reduced. It is assumed that C1 had the best inhibitory effect on *α*‐amylase, which may be due to the fact that C1 contained more flavonoids. During the purification process from D1 to S1, saponins were significantly reduced, while flavonoids did not change. According to this, S1 had the best inhibitory effect on *α*‐glucosidase, which may also be due to the existence of flavonoids. These findings provide an important basis for an in‐depth understanding of the hypoglycemic mechanism of phenolic extracts from *L. barbarum* in Ningxia. Our data showed that the composition of compounds, particularly flavonoids, contributed to the hypoglycemic potential of these extracts. Different components exhibit distinct effects on enzyme activity, warranting further investigation.

## 4 Discussion

Our study reported the phenolic extracts of *L. barbarum* and emphasized their comprehensive chemical analysis and enzymatic inhibitory activities, revealing their role in hypoglycemic potential. Our data specify the decreasing yield of phenolic compounds with the progression of the extraction and purification processes, which is consistent with previous studies on phenolic extraction efficiency (Wen et al. 2017). These variations in C1, D1, and S1 indicate the importance of extraction methods and purification steps. Particularly, the highest TPC in C1 and the highest TFC in S1 propose that different extraction conditions may favor the retention of specific classes of bioactive compounds. The observed decline in phenolic yields during progressive extraction/purification (C1 → D1 → S1) mirrors trends reported for other *L. barbarum* studies (Wen et al. 2017; Wang et al. 2022), but with critical distinctions: C1's high TPC (77.41% *α*‐amylase inhibition) aligns with *L. barbarum* fruit studies (Lam et al. 2024), where crude extracts retained flavonoid‐rich fractions. S1's *α*‐glucosidase inhibition (85.17%) correlates with purified saponins in *L. barbarum* leaves (Lai et al. 2024), suggesting tissue‐specific bioactive partitioning. Notably, the selective loss of flavonoids/phenolic amides (C1 → D1) and saponins (D1 → S1) underscores *L. barbarum* 's unique chemical sensitivity to processing—a phenomenon less pronounced in unrelated species. This specificity reinforces the need for *L. barbarum‐optimized* protocols, as generic phenolic extraction methods may overlook key bioactive fractions (Wang et al. 2022; Lam et al. 2024).

Based on the enzymatic inhibitory activities of the extracts, our data presented their critical roles in carbohydrate digestion and glucose absorption. The superior inhibition of *α*‐amylase by C1 (77.41%) and *α*‐glucosidase by S1 (85.17%) aligns with the hypothesis that flavonoids and other phenolic compounds contribute to these effects; flavonoids likely contributed to the superior inhibition by C1 on *α*‐amylase and similarly supported the inhibitory effect of S1 on *α*‐glucosidase. The correlation between the chemical composition of the extracts and their enzymatic inhibitory activities emphasizes the importance of understanding the structure–function relationships of these compounds, which could inform the development of more effective hypoglycemic agents (Lai et al. 2024). Altogether, this study contributes to the hypoglycemic potential of *L. barbarum* phenolic extracts, emphasizing the role of flavonoids and other phenolic compounds in inhibiting key enzymes involved in carbohydrate metabolism. Future research is warranted to unravel the underlying mechanisms and improve the extraction processes for the development of novel functional foods or pharmaceuticals targeting glycemic control.

## 5 Conclusion

This study employed an advanced analytical platform, UPLC‐Q‐Orbitrap‐MS, combined with Compound Discover software, which allowed the identification of 78 components, including 23 flavonoids and 15 phenolic acids from crude extract C1 of *L. barbarum* fruits. Phenolic extracts of *L. barbarum* with varying purities exhibited a promising hypoglycemic potential, with a higher inhibitory effect on *α*‐glucosidase compared to *α*‐amylase. However, at the same concentration, their inhibitory activity was lower than that of acarbose; the weaker inhibitory activity compared to prior studies could be attributed to the relatively low purity of the extracts. Compound P1 demonstrated a promising hypoglycemic effect. We demonstrated that purification significantly alters the composition and bioactivity of wolfberry polyphenols. These findings contribute significantly to understanding the characteristics and hypoglycemic applications of *L. barbarum* phenolic extracts from Ningxia, emphasizing the importance of advanced purification technology in understanding their composition and bioactivities.

## Author Contributions

**Qian Ma:** writing – original draft (equal). **Linwu Ran:** resources (equal). **Lu Lu:** writing – review and editing (equal). **Lutao Zhang:** writing – review and editing (equal). **Kiran Thakur:** writing – review and editing (equal). **Zhaojun Wei:** writing – review and editing (equal). **Jia Mi:** writing – review and editing (equal). **Yamei Yan:** writing – review and editing (equal).

## Conflicts of Interest

The authors declare no conflicts of interest.

## Data Availability

All data generated or analyzed during this study are included in this published article.

## References

[fsn371077-bib-0001] Abdelli, I. , N. Benariba , and S. Adjdir . 2021. “In Silico Evaluation of Phenolic Compounds as Inhibitors of *α*‐Amylase and *α*‐Glucosidase.” Journal of Biomolecular Structure & Dynamics 3, no. 39: 816–822. 10.1080/07391102.2020.1718553. 31955660

[fsn371077-bib-0002] Adhikari, A. , B. Adhikari , D. Shrestha , R. J. Tharu , N. Gyawali , and H. R. Paudel . 2023. “Antioxidant, *α*‐Glucosidase, and *α*‐Amylase Inhibition Activities of Erythropalum Scandens Blume.” Journal of Chemistry 2023: 1–4. 10.1155/2023/9889113.

[fsn371077-bib-0003] Blesso, L. , and J. Christopher . 2024. “Beneficial Effects of Phenolic Compounds: Native Phenolic Compounds vs Metabolites and Catabolites.” Critical Reviews in Food Science and Nutrition 64, no. 25: 9113–9131. 10.1080/10408398.2023.2208218. 37140183

[fsn371077-bib-0004] Cai, H. , X. Yang , Q. Cai , B. Ren , H. Qiu , and Z. Yao . 2017. “ *Lycium barbarum* L. Polysaccharide (LBP) Reduces Glucose Uptake via Down‐Regulation of SGLT‐1 in Caco_2_ Cell.” Molecules 22, no. 2: 341. 10.3390/molecules22020341. 28241438 PMC6155582

[fsn371077-bib-0005] Chu, M. J. , X. M. Liu , N. Yan , F. Z. Wang , Y. M. Du , and Z. F. Zhang . 2018. “Partial Purification, Identification, and Quantitation of Antioxidants From Wild Rice (*Zizania latifolia*).” Molecules 23: 112782. 10.3390/molecules23112782. PMC6278310 30373196

[fsn371077-bib-0006] De Oliveira Raphaelli, C. , E. Dos Santos Pereira , T. M. Camargo , et al. 2019. “Apple Phenolic Extracts Strongly Inhibit *α*‐Glucosidase Activity.” Plant Foods for Human Nutrition 74, no. 3: 430–435. 10.1007/s11130-019-00757-3. 31302831

[fsn371077-bib-0007] Di, L. C. , F. Colombo , S. Biella , C. Stockley , and P. Restani . 2021. “Polyphenols and Human Health: The Role of Bioavailability.” Nutrients 13, no. 1: 273. 10.3390/nu13010273. 33477894 PMC7833401

[fsn371077-bib-0008] Donno, D. , M. D. Mellano , E. Raimondo , A. K. Cerutti , Z. Prgomet , and G. L. Beccaro . 2016. “Influence of Applied Drying Methods on Phytochemical Composition in Fresh and Dried Goji Fruits by HPLC Fingerprint.” European Food Research and Technology 242, no. 11: 1961–1974. 10.1007/s00217-016-2695-z.

[fsn371077-bib-0009] Echouffo, T. , B. Justin , and R. Garg . 2017. “Management of Hyperglycemia and Diabetes in the Emergency Department.” Current Diabetes Reports 17, no. 8: e0883‐2. 10.1007/s11892-017-0883-2. 28646357

[fsn371077-bib-0010] Figueiredo‐González, M. , C. Grosso , P. Valentão , and P. B. Andrade . 2016. “ *α*‐Glucosidase and *α*‐Amylase Inhibitors From *Myrcia* spp.: a Stronger Alternative to Acarbose?” Journal of Pharmaceutical and Biomedical Analysis 118: 322–327. 10.1016/j.jpba.2015.10.042. 26590699

[fsn371077-bib-0011] Güvenalp, Z. , H. özbek , B. Dursunoğlu , et al. 2017. “ *α*‐Amylase and *α*‐Glucosidase Inhibitory Activities of the Herbs of *Artemisia dracunculus* L. and Its Active Constituents.” Medicinal Chemistry Research 26, no. 12: 3209–3215. 10.1007/s00044-017-2014-7.

[fsn371077-bib-0012] Hao, W. , S. F. Wang , J. Zhao , and S. P. Li . 2020. “Effects of Extraction Methods on Immunology Activity and Chemical Profiles of *Lycium barbarum* Polysaccharides.” Journal of Pharmaceutical and Biomedical Analysis 185: 113219. 10.1016/j.jpba.2020.113219. 32145536

[fsn371077-bib-0013] He, D. J. , Y. Huang , and A. Ayupbek . 2010. “Separation and Purification of Flavonoids From Blackcurrant Leaves by High‐Speed Counter Current Chromatography and Preparative Hplc.” Journal of Liquid Chromatography & Related Technologies 33: 615–628. 10.1002/bmc.5579. 20454590 PMC2863331

[fsn371077-bib-0014] Islam, T. , X. M. Yu , T. S. Badwal , and B. J. Xu . 2017. “Comparative Studies on Phenolic Profiles, Antioxidant Capacities and Carotenoid Contents of Red Goji Berry (*Lycium barbarum*) and Black Goji Berry (*Lycium ruthenicum*).” Chemistry Central Journal 11: 59. 10.1186/s13065-017-0287-z. 29086843 PMC5483215

[fsn371077-bib-0015] Katanić, S. J. S. , N. Mićanović , N. Grozdanić , et al. 2022. “Polyphenolic Profile, Antioxidant and Antidiabetic Potential of Medlar ( *Mespilus germanica* L.) Blackthorn ( *Prunus spinosa* L.) and Common Hawthorn ( *Crataegus monogyna* Jacq.) Fruit Extracts From Serbia.” Horticulturae 8, no. 11: 1053. 10.3390/horticulturae8111053.

[fsn371077-bib-0016] Lai, M. , H. Chen , X. Liu , et al. 2024. “Exploration of Phenolic Profile From Mangosteen ( *Garcinia mangostana* L.) Pericarp and Their Contribution to Inhibitory Effects on *α*‐Amylase and *α*‐Glucosidase.” LWT 203: 116350. 10.1016/j.lwt.2024.116350.

[fsn371077-bib-0017] Lam, T. , N. N. Tran , L. D. Pham , et al. 2024. “Flavonoids as Dual‐Target Inhibitors Against *α*‐Glucosidase and *α*‐Amylase: A Systematic Review of In Vitro Studies.” Natural Products and Bioprospecting 14: 1. 10.1007/s13659-023-00424-w4. 38185713 PMC10772047

[fsn371077-bib-0018] Li, Q. W. , R. Zhang , Z. Q. Zhou , et al. 2019. “Phenylpropanoid Glycosides From the Fruit of *Lycium barbarum* L. and Their Bioactivity.” Phytochemistry 164: 60–66. 10.1016/j.phytochem.2019.04.017. 31096077

[fsn371077-bib-0019] Lu, W. Y. , Q. Q. Jiang , H. M. Shi , et al. 2014. “Partial Least‐Squares‐Discriminant Analysis Differentiating Chinese Wolfberries by UPLC–MS and Flow Injection Mass Spectrometric (FIMS) Fingerprints.” Journal of Agricultural and Food Chemistry 62, no. 37: 9073–9080. 10.1021/jf502156n. 25152955

[fsn371077-bib-0020] Lucia, M. , L. Allison , J. Olivia , and F. Michel . 2020. “Natural Phenolic Compounds and Derivatives as Potential Antimalarial Agents.” Planta Medica 86, no. 9: 585–618. 10.1055/a-1148-9000. 32325510

[fsn371077-bib-0021] Lv, H. , C. C. Xing , M. D. Gao , et al. 2017. “Q‐TOF/MSE Analysis of *Lycium barbarum* Polyphenols From Ningxia and Its Effect on Cellular Antioxidant Capacity.” Journal of Nuclear Agricultural 2, no. 31: 298–306.

[fsn371077-bib-0022] Ma, R. X. , X. Z. Sun , C. Yang , and Y. L. Fan . 2023. “Integrated Transcriptome and Metabolome Provide Insight Into Flavonoid Variation in Goji Berries ( *Lycium barbarum* L.) From Different Areas in China.” Plant Physiology and Biochemistry 199: 107–722. 10.1016/j.plaphy.2023.107722. 37150012

[fsn371077-bib-0023] Ma, Y. L. , Y. Wang , Z. F. Wu , et al. 2023. “Exploring the Effect of In Vitro Digestion on the Phenolics and Antioxidant Activity of *Lycium barbarum* Fruit Extract.” Food Bioscience 51: 102255. 10.1016/j.fbio.2022.102255.

[fsn371077-bib-0024] Magana‐Barajas, E. , G. V. Buitimea‐Cantua , A. Hernandez‐Morales , V. Torres‐Pelayo , J. Vazquez‐Martinez , and N. E. Buitimea‐Cantua . 2021. “In Vitro Alpha‐Amylase and Alpha‐Glucosidase Enzyme Inhibition and Antioxidant Activity by Capsaicin and Piperine From *Capsicum chinense* and *Piper nigrum* Fruits.” Journal of Environmental Science and Health. Part. B 56, no. 3: 282–291. 10.1080/03601234.2020.1869477. 33397190

[fsn371077-bib-0025] Niu, Y. H. , J. L. Liao , H. T. Zhou , C. C. Wang , L. Wang , and Y. L. Fan . 2022. “Flavonoids From *Lycium barbarum* Leaves Exhibit Anti‐Aging Effects Through the Redox‐Modulation.” Molecules 27, no. 15: 4952. 10.3390/molecules27154952. 35956901 PMC9370597

[fsn371077-bib-0026] Onyancha, J. M. , G. A. Moriasi , and E. N. Mandela . 2023. “Nutrient, Non‐Nutrient, Antioxidant Activity, and Fourier Transform Infrared Analysis of Kenyan Indigenous Edible Leafy Vegetables From *Launaea cornuta* (Hochst ex Oliv and Hiern).” African Health Sciences 23, no. 4: 524–536. 10.4314/ahs.v23i4.56. 38974288 PMC11225461

[fsn371077-bib-0027] Qian, D. , J. L. Chen , C. J. S. Lai , et al. 2020. “Dicaffeoyl Polyamine Derivatives From Bitter Goji: Contribution to the Bitter Taste of Fruit.” Fitoterapia 143: 104543. 10.1016/j.fitote.2020.104543. 32151640

[fsn371077-bib-0028] Qian, D. , J. Xi , Y. L. Nie , et al. 2023. “Chemical Characterization of Dicaffeoyl Polyamine Derivatives to Understand Specific Secondary Metabolites in Goji Berry.” Journal of Food Composition and Analysis 122: 105434. 10.1016/j.jfca.2023.105434.

[fsn371077-bib-0029] Shang, Y. F. , T. H. Zhang , K. Thakur , et al. 2022. “HPLC‐MS/MS Targeting Analysis of Phenolics Metabolism and Antioxidant Activity of Extractions From *Lycium barbarum* and Its Meal Using Different Methods.” Food Science and Technology 42: 71022. 10.1590/fst.71022.

[fsn371077-bib-0030] Souza, P. M. , P. M. Sales , L. A. Simeoni , E. C. Silva , D. Silveira , and P. O. Magalhaes . 2012. “Inhibitory Activity of Alpha‐Amylase and Alpha‐Glucosidase by Plant Extracts From the Brazilian Cerrado.” Planta Medica 78, no. 4: 393–399. 10.1055/s-0031-1280404. 22134849

[fsn371077-bib-0031] Taniguchi, M. , C. A. Larocca , J. D. Bernat , and J. S. Lindsey . 2023. “Digital Database of Absorption Spectra of Diverse Flavonoids Enables Structural Comparisons and Quantitative Evaluations.” Journal of Natural Products 86, no. 4: 1087–1119. 10.1021/acs.jnatprod.2c00720. 36848595

[fsn371077-bib-0032] Wang, S. H. , Q. Y. Du , X. L. Meng , and Y. Zhang . 2022. “Natural Polyphenols: A Potential Prevention and Treatment Strategy for Metabolic Syndrome.” Food & Function 13, no. 19: 9734–9753. 10.1039/D2FO01552H. 36134531

[fsn371077-bib-0033] Wang, X. H. , and J. P. Wang . 2019. “Effective Extraction With Deep Eutectic Solvents and Enrichment by Macroporous Adsorption Resin of Flavonoids From *Carthamus tinctorius* L.” Journal of Pharmaceutical and Biomedical Analysis 176: 112804. 10.1016/j.jpba.2019.112804. 31408754

[fsn371077-bib-0034] Wen, L. , Y. L. Lin , R. M. Lv , et al. 2017. “An Efficient Method for the Preparative Isolation and Purification of Flavonoids From Leaves of *Crataegus pinnatifida* by HSCCC and Pre‐HPLC.” Molecules 22, no. 5: 767. 10.3390/molecules22050767. 28486427 PMC6153923

[fsn371077-bib-0035] Wojdyło, A. , P. Nowicka , and P. Bąbelewski . 2018. “Phenolic and Carotenoid Profile of New Goji Cultivars and Their Anti‐Hyperglycemic, Anti‐Aging and Antioxidant Properties.” Journal of Functional Foods 48: 632–642. 10.1016/j.jff.2018.07.061.

[fsn371077-bib-0036] Wu, G. C. , R. B. Mao , Y. R. Zhang , et al. 2022. “Study on the Interaction Mechanism of Virgin Olive Oil Polyphenols With Mucin and *α*‐Amylase.” Food Bioscience 47: 101673. 10.1016/j.fbio.2022.101673.

[fsn371077-bib-0037] Wu, T. , H. Y. Lv , F. Z. Wang , and Y. Wang . 2016. “Characterization of Polyphenols From *Lycium ruthenicum* Fruit by UPLC‐Q‐TOF/MSE and Their Antioxidant Activity in Caco‐2 Cells.” Journal of Agricultural and Food Chemistry 64, no. 11: 2280–2288. 10.1021/acs.jafc.6b00035. 26963650

[fsn371077-bib-0038] Xin, Z. Q. , S. S. Ma , D. B. Ren , et al. 2018. “UPLC‐Orbitrap–MS/MS Combined With Chemometrics Establishes Variations in Chemical Components in Green Tea From Yunnan and Hunan Origins.” Food Chemistry 266: 534–544. 10.1016/j.foodchem.2018.06.056. 30381222

[fsn371077-bib-0039] Xu, L. N. , Y. Li , Y. Dai , and J. Y. Peng . 2018. “Natural Products for the Treatment of Type 2 Diabetes Mellitus: Pharmacology and Mechanisms.” Pharmacological Research 130: 451–465. 10.1016/j.phrs.2018.01.015. 29395440

[fsn371077-bib-0040] Yang, R. F. , C. Zhao , X. Chen , S. W. Chan , and J. Y. Wu . 2015. “Chemical Properties and Bioactivities of Goji ( *Lycium barbarum* ) Polysaccharides Extracted by Different Methods.” Journal of Functional Foods 17: 903–909. 10.1016/j.jff.2015.06.045.

[fsn371077-bib-0041] Yang, Y. H. , Q. M. Liang , B. Zhang , et al. 2024. “Adsorption and Desorption Characteristics of Flavonoids From White Tea Using Macroporous Adsorption Resin.” Journal of Chromatography A 1715: 464–621. 10.1016/j.chroma.2023.464621. 38198876

[fsn371077-bib-0042] Yuan, Y. , J. L. Xiang , B. L. Zheng , et al. 2022. “Diversity of Phenolics Including Hydroxycinnamic Acid Amide Derivatives, Phenolic Acids Contribute to Antioxidant Properties of Proso Millet.” LWT 154: 112611. 10.1016/j.lwt.2021.112611.

[fsn371077-bib-0043] Zhang, G. , S. S. Chen , W. Zhou , et al. 2018. “Rapid Qualitative and Quantitative Analyses of Eighteen Phenolic Compounds From *Lycium ruthenicum* Murray by UPLC‐Q‐Orbitrap MS and Their Antioxidant Activity.” Food Chemistry 269: 150–156. 10.1016/j.foodchem.2018.06.132. 30100417

[fsn371077-bib-0044] Zhang, X. Y. , J. L. Wu , L. Qin , et al. 2022. “Separation and Purification of Two Saponins From Paris Polyphylla Var. Yunnanensis by a Macroporous Resin.” Molecules 27, no. 19: 6626. 10.3390/molecules27196626. 36235164 PMC9570678

[fsn371077-bib-0046] Zhou, Z. Q. 2017. “Study on the Active Ingredients of *Lycium barbarum* Against Alzheimer's Disease.” https://kns.cnki.net/kcms2/article/abstract?v.

[fsn371077-bib-0045] Zhou, Z. Q. , H. X. Fan , R. R. He , et al. 2016. “Lycibarbarspermidines A–O, New Dicaffeoylspermidine Derivatives From Wolfberry, With Activities Against Alzheimer's Disease and Oxidation.” Journal of Agricultural and Food Chemistry 64, no. 11: 2223–2237. 10.1021/acs.jafc.5b05274. 26953624

[fsn371077-bib-0047] Żołnierczyk, A. K. , S. Ciałek , M. Styczyńska , and M. Oziembłowski . 2021. “Functional Properties of Fruits of Common Medlar ( *Mespilus germanica* L.) Extract.” Applied Sciences 11, no. 16: 7528. 10.3390/app11167528.

